# An evaluation of gender equity in different models of primary care practices in Ontario

**DOI:** 10.1186/1471-2458-10-151

**Published:** 2010-03-23

**Authors:** Simone Dahrouge, William Hogg, Meltem Tuna, Grant Russell, Rose Anne Devlin, Peter Tugwell, Elisabeth Kristjansson

**Affiliations:** 1 C.T. Lamont Primary Health Care Research Centre, Élisabeth Bruyère Research Institute, 43 Bruyère Street, Ottawa, Ontario, Canada; 2 University of Ottawa, Department of Family Medicine, 43 Bruyère St, Ottawa, Ontario, Canada; 3 University of Ottawa, Department of Epidemiology and Community Medicine, 451 Smyth Road, Ottawa, Ontario, Canada; 4 University of Ottawa, Institute of Population Health, 1 Stewart St, Room 300, Ottawa, Ontario, Canada

## Abstract

**Background:**

The World Health Organization calls for more work evaluating the effect of health care reforms on gender equity in developed countries. We performed this evaluation in Ontario, Canada where primary care models resulting from reforms co-exist.

**Methods:**

This cross sectional study of primary care practices uses data collected in 2005-2006. Healthcare service models included in the study consist of fee for service (FFS) based, salaried, and capitation based. We compared the quality of care delivered to women and men in practices of each model. We performed multi-level, multivariate regressions adjusting for patient socio-demographic and economic factors to evaluate vertical equity, and adjusting for these and health factors in evaluating horizontal equity. We measured seven dimensions of health service delivery (e.g. accessibility and continuity) and three dimensions of quality of care using patient surveys (n = 5,361) and chart abstractions (n = 4,108).

**Results:**

Health service delivery measures were comparable in women and men, with differences ≤ 2.2% in all seven dimensions and in all models. Significant gender differences in the health promotion subjects addressed were observed. Female specific preventive manoeuvres were more likely to be performed than other preventive care. Men attending FFS practices were more likely to receive influenza immunization than women (Adjusted odds ratio: 1.75, 95% confidence intervals (CI) 1.05, 2.92). There was no difference in the other three prevention indicators. FFS practices were also more likely to provide recommended care for chronic diseases to men than women (Adjusted difference of -11.2%, CI -21.7, -0.8). A similar trend was observed in Community Health Centers (CHC).

**Conclusions:**

The observed differences in the type of health promotion subjects discussed are likely an appropriate response to the differential healthcare needs between genders. Chronic disease care is non equitable in FFS but not in capitation based models. We recommend that efforts to monitor and address gender based differences in the delivery of chronic disease management in primary care be pursued.

## Background

Primary care is the foundation of the Canadian health care system. Recent Canadian [1] and international policy recommendations [2] have emphasised the need for investments in primary health care systems to improve efficiencies and reduce inequities. There is convincing evidence that stronger primary health care systems can reduce disparities in health between regions [3]. However few studies have investigated whether the organization of the primary care system impacts on equitable care across individuals.

Evaluations of equity can be seen from two perspectives. Vertical equity addresses whether treatment is preferentially delivered to those with greater health needs, while horizontal equity considers whether there is the provision of equal treatment for equivalent needs [4]. For example, vertical equity would dictate that an individual with multiple health problems should receive greater care than a healthy individual, while horizontal equity would require that two individuals with similar health status receive similar care levels regardless, for example, of their socio-economic status. Both paradigms are important to consider.

Ontario, Canada's largest province, organises primary care practices under different "models of care", most of which emerged following a series of provincial initiatives over the past four decades that aim to build a more accessible, patient oriented system and eliminate the barriers inherent in the traditional Fee For Service (FFS) model [5]. The first attempts at reforming primary care came with the introduction of Community Health Centres (CHC) and Health Service Organizations (HSO) in the 1970s. CHCs are a community orientated model in which providers are salaried. Integral in many CHCs' mission statement are the notions of social justice and equity [6-8]. HSO is a capitation based model; a payment structure that offers a fixed monthly remuneration fee based on the age and sex of enrolled patients for basic primary care services, regardless of the number of services provided [9]. A second capitation model which also offered additional accessibility and comprehensiveness incentives, Family Health Networks, (FHNs) was established in the early 2000s. Because compensation in capitation based practices is dissociated from visit number, proponents of this type of remuneration approach expect care to be more equitably dispensed; in response to need with reduced concerns over output. In fact, primary care capitation based funding was recently introduced in New Zealand [10] and Thailand [11] in part in an effort to reduce inequities. Today, capitation based practices and CHCs serve approximately 40%, and 3%, respectively of the population in Ontario.

Some studies have evaluated the impact of these reforms on the quality of the care delivered, [12,13] but none have studied their impact on the equitable delivery of care. In a recent review, the World Health Organization calls for more work evaluating the effect of health care reforms on gender equity [14]. This study evaluates whether gender differences in the primary care experience in each model exist and whether the extent of gender differences between models differs. This study is part of a larger evaluation exploring the impact of primary care reforms on equity.

## Methods

### Design

This study uses data from a cross sectional study conducted in Ontario, Canada in 2005-6; the Comparison of Models in Primary Care (COMP-PC) [15]. Data were gathered from primary care practices, providers (family physicians and nurse practitioners) and patients receiving care at these practices. A detailed description of the overall study methodology is available elsewhere. The study was approved by the Ottawa Hospital Research Ethics Board.

### Sample

The COMP-PC study evaluated the performance of FFS, CHC, HSO, and FHN across a number of domains. Table 1 summarizes key features of each model. The study had a recruitment strategy stratified by model. Randomly selected eligible FFS practices (n = 155) and all (n) known and eligible CHC (51), HSO (65), and FHN (94) practices were approached for participation. Recruitment was closed when 35 practices per model agreed to participate or when time constraints didn't permit further recruitment.

**Table 1:** Ontario's main primary care models in 2005/2006.

	Community Health Centre (CHC)	Fee for service (FFS)		Family Health Network (FHN)	Health Service Organization (HSO)
		**Traditional** **Fee for Service**	**Family Health Groups (FHG)^1^**		

**Year introduced**	1970s	-	2004	2001	1970s

**Group size**	Groups practice - Unspecified size	1 Physician	Minimum 3	Minimum 3	Minimum 3

**Physician remuneration**	Salary	FFS	FFS and incentives	Capitation^2 ^with a 10% FFS component, and incentives	Capitation^b^ and incentives

**Patient enrolment**	Required No roster size limit	Not required	Required No roster size limit	Required Disincentive to enrol >2,400^3^	Required Disincentive to enrol >2,400^3^

**Access**	No specified requirements	No specified requirements	THAS^4^ Extended hours^5^	THAS Extended hours^5^ Access bonus^6^	THAS Extended hours^5^ Access negation^7^

**Multi-disciplinarity^8^**	Significant	None	None	Some	Some

**Assistance for Information Technology**	Some	None	None	Yes	None

**Objectives/Priorities**	Responsiveness to population needs, multi-disciplinarity, prevention, focus on underserved, equity community governed	-	Accessibility	Accessibility, comprehensiveness, doctor-nurse collaboration, use of technology	Responsiveness to population needs, multi-disciplinarity, health promotion, cost effectiveness

^1^Late in 2004, the Ontario Ministry of Health (MOH) created a new model of care, the FHG, to which FFS practices could transition. Family Health Groups (FHG) needed to comprise three or more family physicians practicing together. These physicians need not be located in the same physical office space, but must be within reasonable distance of each other. FFS practices converted to this new model quickly, so that by early 2006 most FFS practices had become FHGs, and it became evident that the great majority would transition by the year end.

^2^Under capitation remuneration, family physicians received a fixed monthly fee per patient enrolled, independent of the number of visits made to the practice by that patient. The capitation fee is based on the enrolled patient sex and age. FHN physicians receive an additional 10% of the FFS structure for each visit. The later is principally intended to allow for a better monitoring of the services delivered.

^3^The base capitation rate is reduced to 50% for patients enrolled to a provider with a practice size exceeding 2,400

^4 ^THAS = Telephone Health Advisory Service - A 24 hrs/7 days a week patient telephone advisory service available to enrolled patients.

^5^Each physician is required to provide at least one 3 hour session outside regular hours (evening/week end) per week (up to 5 sessions per group/network/organization)

^6 ^An incentive bonus that is reduced in relation to the number of visits patients make to non-specialists outside the FHN.

^7 ^A penalty incurred from the capitation fee for visits patients make to non-specialists outside the FHN.

^8 ^Multi-disciplinarity refers to the presence of allied health workers (e.g. dietician, social worker, and pharmacist), excluding nursing staff, but including nurse practitioners.

Informed by the Ontario Medical Association's "Comparison of Models" table - https://www.oma.org/PC/PCRComparisonJan0807.pdf (PCRComparisonJan0807.pdf)

### Data collection

The study recruited 137 practices, surveyed 5,361 patients in the waiting room sequentially (response rate: 82%) as they presented for their appointment ("index visit") and performed a review of 4,108 randomly/systematically selected charts. Those patients not participating in the survey most frequently sited a lack of time to participate. Surveyed patients (30-50/practice) were required to be under the care of one of the participating providers, aged 18 years or older, not severely ill or cognitively impaired, and able to communicate in English or French either directly or through a translator. Charts reviewed were limited to those of patients ages 18 years and older who had been with the practice at least two years.

### Instruments

Surveys were adapted from the Primary Care Assessment Tool (PCAT)-Adult edition[16,17] and supplemented with two additional scales [18,19]. The patient survey was divided into two sections. The first was completed in the waiting room before the encounter with the provider and captured socio-demographic and economic information, and elicited patient's experience on the quality of health service delivery. The second was completed after the appointment with the provider and captured visit-specific information, including a measure of health promotion activity. The survey tool was available in English and French [20]. Translators were used in practices in which a significant proportion of the population was expected to have limited or no English or French language skills.

The chart audit collected patient sex, age, and insurance status and measured preventive care and chronic disease management by comparing chart documentation of these activities against recommended guidelines. We measured the provider's recommendation for a manoeuvre rather than patient compliance, and coded it as "done" if it was performed or recommended/discussed even it not done.

### Performance measures

We assessed performance across seven dimensions of health service delivery and three dimensions of technical quality of care (Table 2). The technical quality of care scales are further described in related manuscripts [[12,21], Dahrouge S, Hogg W, Russell G, Tuna M, Geneau R, Muldoon L *et al*.: The Impact of Remuneration and Organizational Factors on Prevention Activity in Primary Care: A cross sectional study. *Submitted*].

**Table 2:** Scales for the measurement of performance

Quality of Health Care Service Delivery^a ^(items in the scale, categories in the likert scale of each item)		Source of data	Overall score ranges^c^
Access	*First contact accessibility (4, 4)*	Patient survey	74% - 83%
	*First contact utilization (3, 4)*	Patient survey	96% - 98%

Patient-Provider	*Humanism (8, 7)*	Patient survey	90% - 91%
Relationship	*Trust (10, 5)*	Patient survey	87% - 88%
	*Cultural competency (3, 4)*	Patient survey	83% - 85%
	*Family centeredness (3, 4)*	Patient survey	89% - 90%

Continuity	*Ongoing care (4, 4)*	Patient survey	85% - 90%

**Technical Quality of Clinical Care Delivery^b^**- Adherence to recommended guidelines (items in the scale)			

Health Promotion	*Healthy lifestyle counseling (7)*	Patient survey	46% - 59%

Prevention	*Preventive care (6)*	Chart audit	52% - 68%

Chronic Disease Management	*Chronic disease management (9)*	Chart audit	60% - 72%

^a ^All health care service delivery scales are based on the PCAT[16,17], except for the Humanism, [42] and Trust[43] scales.

A respondent's scale was included only if at least 50% of its items contained a response. Performance scores for each health service delivery scale were derived by summing the individual item scores and normalizing these to a percentage. For example, for first contact accessibility, the sum of the scores for the four questions, each on a likert scale of 1-4, is divided by 16

^b^Health promotion and prevention evaluations were based on the Canadian Task Force on Preventive Health Care (CTFPHC) clinical practice guidelines[44]. Chronic disease management was assessed against recommended guidelines accepted in Ontario for the management of the conditions [45-51].

For health promotion, patients were asked to indicate which of 7 subjects were discussed with them on that day's visit. We assessed whether at least one subject was discussed on that visit, and estimated the overall extent of health promotion delivered yearly by multiplying the number of subjects discussed at the index visit by the patient's estimated number of visits to that practice for the year. Preventive care was determined by assessing the performance of 6 indicator manoeuvres in the chart audit. The prevention score was the proportion of preventive manoeuvres for which the individual was eligible that were documented. Finally, chronic disease management was also evaluated by chart audit using 2-4 indicators in each of three conditions (Diabetes, Coronary Artery Disease and Congestive Heart Failure). For each condition the score was derived as for prevention, and the overall chronic disease management score was the average of the individual disease scores.

^c^Indicates the range of scores for each scale in the four models.

### Analysis

#### 1. Identify gender differences

We compared the performance scores for women to those of men while adjusting for potentially confounding factors using multi-level multivariate regressions for all evaluations except chronic disease management. For the latter, too few observations per practices were available to warrant adjusting for clustering effect with multi-level analyses. For analyses relying on patient survey data, we adjusted for patient socio-demographic and economic characteristics (identified as SE in Table 3) in one analysis, and added measures of health (identified as H in Table 3) in the second analysis. The analyses including health factors inform the horizontal equity evaluation, while those in which it was omitted inform the vertical equity evaluation. For analyses relying on chart data, we had inadequate information on health status, and so only conducted analyses adjusted for age, rurality, and insurance status. In all analyses, Age*Gender interactions were considered and used where appropriate. Variable imputation was used to avoid case-wise deletions.

**Table 3:** Profile of patients by gender

Survey patient profile	Men	Women
^1^ **Socio-demographic and economic profile**		

SE Age (mean^‡^, median in years)	53/53	48/47

SE Household income (% under LICO)^‡^	13	19

SE Low education (% with less than high school degree) *	19	16

SE Not speaking English or French at home (%)	1.7	1.9

SE Aboriginal (%)*	0.8	1.6

SE Uninsured (in Canada) (%)	1.6	2.3

SE Not working outside the house (%)	37	26

SE Recent immigrant (< 5 years) (%)	2.0	2.5

SE Rurality index (mean)	13	13

SE Distance from home to practice > 10 km (%)	26	25

**Health status**		

H At least one day with poor mental health in past 30 days (%)^‡^	34	49

H At least one day with poor physical health in past 30 days (%)^‡^	56	62

H At least one day limited by poor mental or physical health in past 30 days (%)*	40	43

H Physical, mental or emotional problem lasting more than one year (%)	43	41

H Self perceived health good-excellent (%)	82	82

H Presence of at least one chronic disease/Number of chronic diseases (%)	74/1.9	73/1.8

** Relationship with the practice**		

Provider is a Nurse Practitioner (%)^‡^	2.1	7.5

Seeing their own provider at that visit (%)	91.5	92.1

Attending the practice for more than 2 years (%)	83	83

Number of visits to the office in previous year (mean^†^, median)	5.8, 4	6.6,4

Main reason for visit - Check up/Chronic problem/Recent problem	35/30/36	36/27/37

** Chart audit patient profile**		

Uninsured in Ontario (%)*	0.7	1.6

Age (mean^‡^, median in years)	49.5/48	46.0/45

Number of visits to the office in previous year (mean^‡^, median)	4.3/3	5.0/4

^1 ^In this column socio-demographic and economic factors used for adjustment in the vertical equity analyses are identified as SE, and health related factors used for adjustment in the horizontal equity analyses are identified as H.

LICO = Low Income Cut off, a measure of household deprivation used by Statistics Canada[52]

The following symbols reflect the significance level * p < 0.05, ^† ^p < 0.01, ^‡ ^= p < 0.001 compared by Pearson Chi Square or independent t-test.

We performed multi-level linear regressions for continuous outcomes using SPSS 16, and multi-level logistic regressions for binary outcomes using the Glimmix procedure in SAS. The analyses were stratified by model. All results shown reflect the effect of being a female compared to being a male.

#### 2. Compare the extent of gender differences between models

The effect sizes (absolute beta values) of the gender variable in each model derived from the regressions performed to meet objective #1 were compared using the t-statistics to evaluate whether models were significantly different in their gender effect.

When meaningful gender differences are observed, we estimated the adjusted performance level for the "typical" women and men. Using the beta coefficients from the regression equation developed to meet objective #1, we calculated the performance level for the "typical" practice patient.

## Results

### Characteristics of the study population

The study population was determined to be adequately representative of its underlying population [15]. There were significant differences in several patient characteristics between genders (Table 3). Notably, women surveyed were significantly more likely to report days with poor mental or physical health and limitations related to these conditions. However, self perceived health was similar in both groups.

### Gender differences in performance

Overall, women reported more visits than men (6.6 vs 5.8, p < 0.01), with adjusted differences (95% confidence interval (CI)) of CHC: +1.0 (-0.7, 2.7); FFS: +0.6 (-0.4, 1.5); FHN: +1.2 (0.6, 1.8); HSO: +0.8 (0.3, 1.3). We found no difference in the reported duration of the index visit between women and men.

#### Health service delivery scales

**Figure 1:**
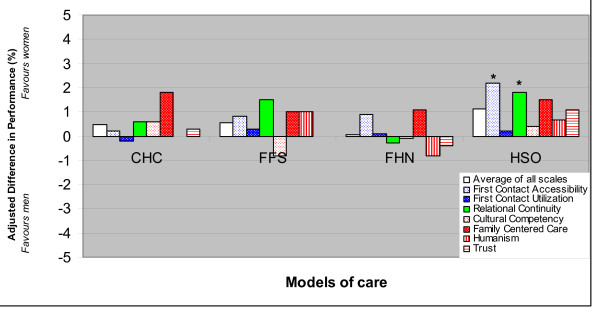
**Health service delivery across gender - Effect of being a woman**. (Adjusted for socio-economic and health status). The number of evaluable patients in each analysis was as follows: First contact accessibility: 5005; First contact utilization: 5272; Cultural competency: 4709; Humanism: 5243; Family centered care: 5097; Trust: 5227; Relational continuity: 5245. The adjusted difference in performance between women and men are shown. The effect is adjusted for patient socio-demographic and economic factors and health status using multi-level linear regression. Statistically significant (p < 0.05) results are indicated by "*". Results of the analyses in which health status were not included are consistent with these results. There were no significant differences in the extent of gender differences in any performance measure across models.

Differences between genders in all health service delivery measures were not clinically meaningful (≤ 2.2%) in the analyses including and excluding health status variables (Figure 1).

#### Technical quality of care scales

##### Health promotion

**Figure 2:**
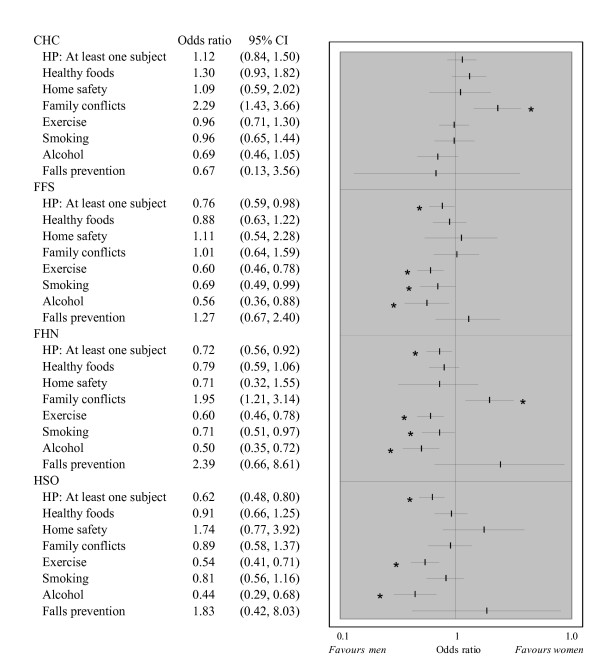
**Health promotion across gender - Odds ratio of women relative to men**. (Adjusted to socio-economic and health status). 4,794 individuals had provided sufficient information to be included in this analysis. The "HP: At least one subject" variable represents the likelihood that at least one health promotion subject was discussed at the index visit. All other variables represent the likelihood that the subject was discussed at the index visit. Odds ratios are adjusted for patient socio-demographic and economic factors and health status. Statistically significant gender differences (p < 0.05) are indicated by "*". Results of the vertical equity analyses in which health status were not included are consistent with these results.

The odds that at least one health promotion item was discussed at the index visit were lower in women in all models but CHCs (Figure 2). However, since women have more frequent yearly visits, the overall estimated number of subjects discussed over a 12 months period was not significantly different in the two groups in any model. We observed significant gender differences in the type of subjects discussed at the index visit. Women were more likely to have discussed family conflicts in CHCs and FHNs. This effect in was significantly larger than in all other models. Men were significantly more likely to have discussed smoking in FFS and FHNs, and exercise and alcohol consumption in all models but CHCs. The gender effect for exercise discussion was significantly larger in FFS, FHN and HSO compared to CHCs.

**Figure 3:**
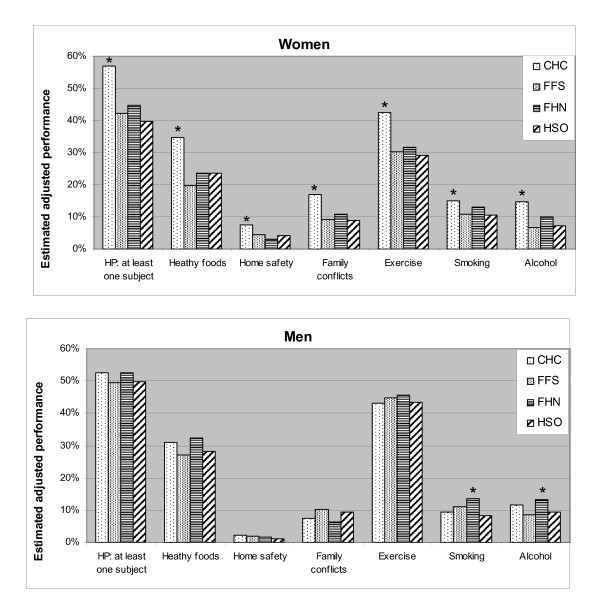
**Adjusted estimated likelihood of a subject being discussed**. (Adjusted for socio-economic and health status). Women were more likely to discuss HP items in CHCs than in any other model. CHCs were statistically superior to all models for all items, except smoking in FHN. Men were usually equally likely to discuss HP items in all models, although men attending FHNs were more likely than those attending HSO to discuss smoking and more likely than those attending FFS to discuss alcohol. The estimated performance for men and women in each model is shown for the "typical" patient; an individual with the most common features: Age 30-49 (except for fall prevention, where it is <75), without a disadvantaged feature (low education, income below low cut off, language barrier, aboriginal status, uninsured), travel distance less than 10 km, not rural, no limitations due to physical or mental health, or problem lasting more than one year, health good-excellent, and the presence of at least one chronic disease. Results of the vertical equity analyses in which health status were not included are consistent with these results. Statistically significant gender differences (p < 0.05) are indicated by "*".

Figure 3 shows the estimated adjusted likelihood of each subject being discussed in the "typical" women and men in each model. Men were not less likely to report discussing smoking, alcohol, or exercise in CHC than in other models. In contrast, women reported HP discussion for virtually all subjects more frequently in CHCs than other models.

##### Preventive care

**Figure 4:**
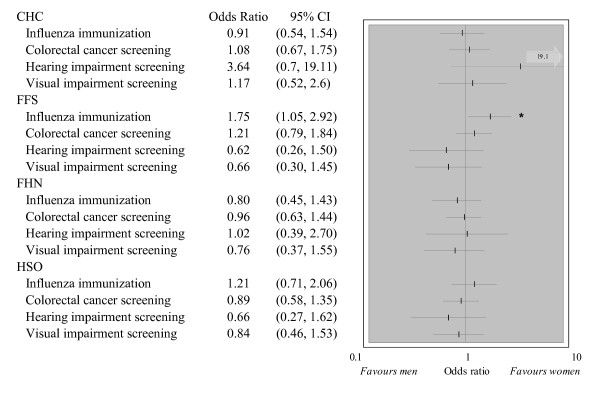
**Individual preventive manoeuvres across genders**. Odds ratios are adjusted for age, insurance status and rurality. The number of patients eligible for individual manoeuvres was: influenza immunization: 1,365; colorectal cancer screening: 1,753; hearing impairment screening: 651; and visual impairment screening: 735. In CHCs, 2 of 31 men while 17 of 67 women 65 years of age or older had a hearing impairment screening. Because of the small number of events amongst men, the odds ratio confidence interval is unstable. Statistically significant gender differences (p < 0.05) are indicated by "*".

The composite prevention score for all 6 manoeuvres in the 3,284 eligible individuals was significantly higher in women than men in all models. The adjusted effect sizes (95% CI) were: CHC 18% (12%, 25%); FFS 21% (15%, 27%); FHN 13% (8%, 19%); HSO 17% (10%, 23%). This was due to the greater adherence to recommended care for the two female specific manoeuvres measured. When these are excluded from the evaluation, the adjusted effect sizes (95% CI) based on the four remaining manoeuvres in the 2,096 patients were: CHC -2% (-10%, 6%); FFS 4% (-3%, 11%); FHN -4% (-11%, 3%); HSO 0% (-6%, 6%). With one exception, there were no significant gender differences in individual manoeuvres (Figure 4).

##### Chronic disease management

**Figure 5:**
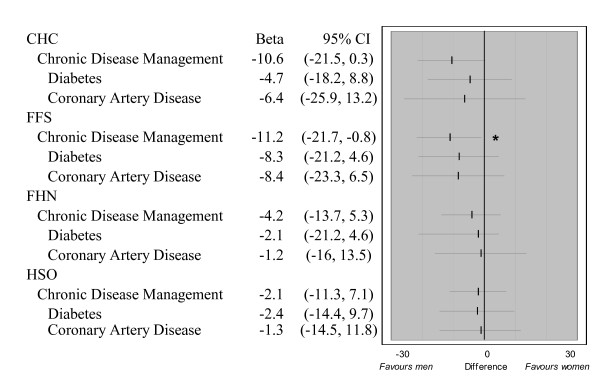
**Overall chronic disease management across gender**. 514 patients had at least one of the three indicator chronic diseases and were included in evaluating CDM; 313 had diabetes, and 273 had CAD. Too few patients had CHF (57) to perform a gender evaluation across models. The gender effect is adjusted for age, insurance status, and rurality. Statistically significant gender differences (p < 0.05) are indicated by "*".

Overall adherence to recommended guidelines for chronic disease management was significantly inferior in women in FFS (difference of -11.2%, 95% CI: -21.7%, -0.8%), and showed a similar trend in CHCs (Figure 5). However, there was no statistically significant difference in the gender effect between models.

**Figure 6:**
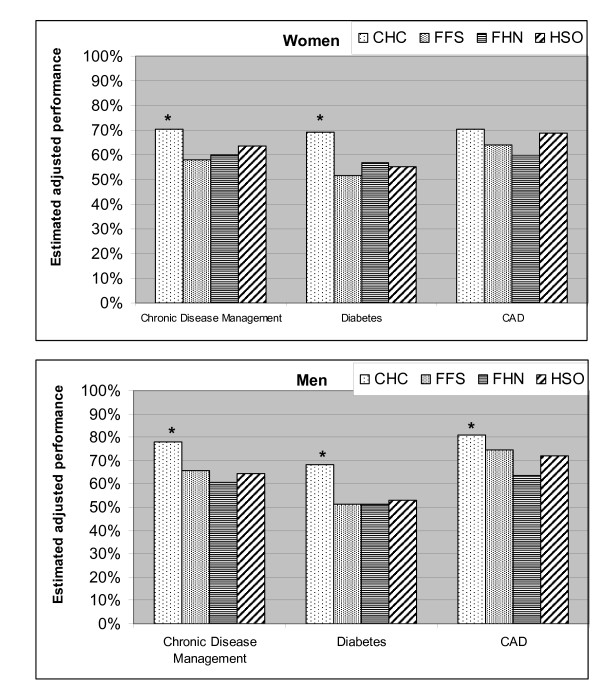
**Adjusted estimated likelihood of a subject being discussed - Horizontal equity**. The estimated performance for men and women in each model is shown for the "typical patient profile", i.e. an individual with the most common features: Age 70 years or older with public health insurance (rurality "0"). Adherence to recommended guidelines in women was highest in CHC than other models for diabetes and for overall chronic disease management. Adherence to recommended guidelines in men was highest in CHC than other models for chronic disease management and diabetes, and was higher in CHC than FHN for CAD. Statistically significant differences (p < 0.05) in equity level between CHC and other models are indicated by "*".

Figure 6 shows the estimated adjusted chronic disease management score in the "typical" women and men in each model. CHCs provided significantly better care to women than other models, while the care received by men was similar for most measures between models. The chronic disease management score in women was not significantly lower in FFS than FHN or HSO. Despite showing a tendency for gender disparity, CHCs were superior to other models in the delivery of chronic disease care for men and women.

## Discussion

Women attending FFS practices were significantly less likely to have received chronic disease care according to recommended guidelines. We observed a similar trend in CHCs but not in capitation based practices. We also found differences in the health promotion topics reported being discussed between women and men, and these differences varied by model. Women were more likely than men to report discussing family conflicts in CHCs and FHNs, whereas men were more likely than women to report discussing smoking in FFS and FHN consultation, and discussing exercise and alcohol consumption in all models but CHCs.

### Health Service Delivery

Consistent with a previous Canadian report, women reported more frequent visits to their primary care practice than men [22]. However, self reported measures of accessibility as well as other dimensions of health service delivery were not meaningfully different in the two groups. We conclude that the delivery of primary care services is equitable across gender in all models.

### Technical quality of care

#### Health promotion

The World Health Organization states that gender equity "... *requires that men and women will be treated equally where they have common needs, and that their differences will be addressed in an equitable manner*." [23] Men are more likely to smoke and abuse alcohol and illicit drugs than women, [24-26] while women are more likely to suffer from family conflicts, [27] suggesting that the gender differences observed are likely an appropriate response to the differential healthcare needs between sexes.

We found the smallest gender gap and best performance for women in CHCs. These results may reflect the focus on health promotion and preventive care integral to CHCs, and the substantially longer visits that would allow time for these activities.

#### Prevention

We observed no significant gender differences in the delivery of colorectal cancer screening and hearing or visual impairment screening in any model but found that men attending FFS were significantly more likely to have been up to date on influenza immunization. Other studies had also found no gender difference in colorectal cancer screening[28] but a higher likelihood of influenza immunization in men receiving care under the Veteran's Health Administration's services, a system that supports both the fee for service and capitation structures [29]. Conclusions about whether gender disparities exist in preventive care is appreciably impacted by the indicators selected. Other studies, as our did, find significantly better preventive scores when conditions specific to women (breast and cervical cancer screening) are included in the overall preventive score, [30] likely because significant investments have been made to promote awareness and compliance for these manoeuvres.

#### Chronic disease management

Our results suggest that gender gaps in the quality of care received may be dependent on the model of care. Women attending FFS practices but not in capitation based models were significantly less likely to have received recommended care for chronic diseases. Because this study captured the provider's intent for processes of care, the results point to a disparate approach in the primary care providers' management of chronic diseases between men and women in FFS practices rather than, say, gender differences in patient compliance to these processes.

Studies using simulated patients with congestive heart failure found men were more likely undergo clinical investigations [31]. Others have found men to be more likely to receive more evidence based cardiovascular preventive care for aspirin prescription, [32,33] triple anti-anginal therapy, [34] beta blocker, [35,36] and angiotensin converting enzyme inhibitors [37-39]. Evidence for diabetic care is less well documented and doesn't show preferential gender treatment [40,41].

FFS is the most common model of care in Ontario, serving nearly 60% of its population. Critics of the FFS model contend that the "per visit" fee structure encourages shorter, problem focused visits, while capitation or salary based remuneration systems should achieve better care because the provider is not penalized for additional time spent on those with greater needs. The results of this evaluation support this notion.

#### Impact of primary care reform on gender equity

Our results suggest that primary care reforms have not had a negative impact on the equitable delivery of primary care across gender. In fact, capitation based practices may provide more equitable chronic disease management and influenza immunization than FFS practices.

#### Strengths and limitations

The survey study population is limited individuals accessing care, and its results cannot be extrapolated to the general population. Because estimates of health service delivery are based on self reported measures, the patient's prior experience and expectation of care, which could plausibly differ by gender, is likely to impact their response.

The evaluation of preventive care and chronic disease management was based on the abstraction of charts. Since these contain very limited patient socio-demographic information we were unable to account for differences in these factors across gender. We also did not capture additional health information to allow us to evaluate whether gender differences in care is related to existing co-morbidities. Finally, we could not evaluate whether patient provider gender concordance is a vehicle to gender disparity.

This "within model" approach to evaluating equity has two advantages. First, it eliminates the effect of differences in the profile of the populations within a model for which one could not adjust. It also allows us to evaluate the impact of the primary care reform initiative that addresses remuneration approach on equity.

## Conclusions

This is the first study to perform an evaluation of that scope of primary care dimensions. We found the experience of health care service delivery to be similar in women and men. The gender differences that we found in the discussion of healthy lifestyle subjects may be an appropriate and efficient response to prioritizing care in response to differential health needs given limited visit time. This study documents inequities in the delivery of chronic disease care in FFS practices but not in capitation based practices. We recommend that efforts to monitor and address gender based differences in the delivery of chronic disease management in primary care be pursued.

## Competing interests

The authors declare that they have no competing interests.

## Authors' contributions

SD conceptualised the analysis, participated in data collection interpreted the data and wrote the initial draft of the manuscript. MT contributed towards methodological and statistical analysis, as well as critically reviewed and edited the manuscript. WH, GR, RAD, EK conceptualized the original study, and along with PT were consulted on the analytical approach, critically reviewed and edited the manuscript. All authors have read and approved the final manuscript.

## Pre-publication history

The pre-publication history for this paper can be accessed here:

http://www.biomedcentral.com/1471-2458/10/151/prepub
